# Tobacco company strategies for maintaining cigarette advertisements and displays in retail chain stores: In-depth interviews with Korean convenience store owners

**DOI:** 10.18332/tid/94829

**Published:** 2018-10-03

**Authors:** Ji-eun Hwang, Yu-mi Oh, Yu-seon Yang, Seon-young Lee, Joung-eun Lee, Sung-il Cho

**Affiliations:** 1 Department of Public Health Science, Graduate School of Public Health, Seoul National University, Seoul, South Korea; 2 Korea Health Promotion Institute, Seoul, South Korea; 3 Gyeonggi Infectious Disease Control Center, Gyeonggi-do, South Korea; 4 Institute of Health and Environment, Seoul National University, Seoul, South Korea

**Keywords:** advertising and promotion, point of sale, tobacco company

## Abstract

**INTRODUCTION:**

This study gathered data from store owners regarding the advertising and display of, and the contractual arrangements and promotional activities related to, the sale of tobacco products in convenience stores.

**METHODS:**

In-depth interviews were conducted with three owners of convenience stores in South Korea: to examine the procedures for managing the sale of tobacco products; for allocating the advertising allowance for such products; and for coordinating the promotional activities of tobacco companies.

**RESULTS:**

All tobacco advertisements and displays in convenience stores are installed and managed in accordance with the contract between the tobacco companies and the convenience store headquarters. The headquarters receives an allowance from the tobacco company in return for maintaining and displaying their product and promotional materials. The headquarters then pays a monthly advertising allowance to each franchisee as an operating subsidy. However, the owners also stated that tobacco companies provide financial incentives directly to them to engage in illegal promotional activities.

**CONCLUSIONS:**

Because tobacco advertisements and displays at convenience stores are related to the profitability of these products, the participants in these relationships have become increasingly entangled. Illegal promotional activities must be monitored to limit tobacco sales and advertising. Furthermore, efforts to ban the advertising and display of tobacco products at the point of sale must be based on the development of policies emerging from an understanding of the roles of the major stakeholders.

## INTRODUCTION

Tobacco advertising and displays at points of sale (POS) maintain smoking habits, interfere with attempts to quit smoking^1^, and are associated with the impulse buying of cigarettes^2,3^. Moreover, these activities influence the initiation of smoking among adolescents, as this group makes frequent visits to retail establishments that sell tobacco and display tobacco advertisements^4,5^. Indeed, frequent exposure to tobacco advertising is associated with higher cigarette brand awareness and increased susceptibility to smoking^6,7^.

For these reasons, Article 13 of the WHO Framework Convention on Tobacco Control (FCTC) stipulates that Parties to the agreement implement comprehensive bans on tobacco advertising, promotion, and sponsorship (TAPS)^8^. That is, Parties are bound to take appropriate legislative actions to prohibit the advertisement of all types of tobacco within 5 years after entry into force of this Convention^8^. Its guidelines also call for a total ban on the display of tobacco products at POS, as such displays directly or indirectly promote the purchase of tobacco products by the public^9^.

Contrary to these global efforts, the international tobacco industries, including Philip Morris International (PMI), British American Tobacco (BAT), Japan Tobacco International (JTI), and Imperial Tobacco Group, are targeting the populations of low-and-middle-income countries, including women and children, in efforts to achieve market expansion^10^. Thus, although the prevalence of smoking is declining globally, its decline in low- and middle-income countries has been more difficult to achieve compared with that in high-income countries, and the number of smokers in the former has not changed^11^. Additionally, tobacco products are being sold at various points of sale^12^ in the absence of restrictions on sellers, on places of sale, and on sales volume. Notably, the global trend in the sales of tobacco products is shifting to small stores that are part of a chain, which are increasingly dominating the domestic distribution market^13^. Indeed, these retail chains are considered major clients of tobacco companies as well as main advertising channels because tobacco companies can advertise in every franchisee after a contract with the headquarters has been signed^14^.

In South Korea, cigarettes can be sold by only those retailers that are designated by the relevant municipal government agencies, but a tobacco retail license is not required^15^. Indeed, cigarettes are sold at convenience stores, including hypermarkets, retail outlets, and kiosks. Recently, however, the number of convenience stores in South Korea, most of which advertise tobacco products, has been increasing^16^. Additionally, the number of cigarette advertisements in convenience stores (an average 20.8 advertisements per store) is higher than that in supermarkets (an average 8.0 advertisements) and small cigarette retailers (an average 3.5 advertisements)^16^. This implies that anyone who visits a convenience store, including adults, children, and adolescents, has intense exposure to tobacco products and advertisements. Tobacco companies have concentrated considerable resources on displays and advertisements placed in POS^17^ because they are important ways to communicate with current and future customers^18^.

In South Korea, existing regulations do not comprehensively ban TAPS. According to the National Health Promotion Act, the display of tobacco products or the posting of tobacco advertising materials in POS is authorized if the displayed or posted advertisement is not visible from the exterior of the store^19^. Violation of this law is punishable by a fine not exceeding 10 million won (about 10 thousand U.S. dollars). Also, according to the Tobacco Business Act, no manufacturer, importer or wholesaler can offer money or goods to a retailer to promote tobacco sales^15^. A person who offers money, goods, or other inducements in violation of the Act can be punished by a fine not exceeding five million won (about five thousand U.S. dollars).

Previous research has shown that a tobacco company and the retailer sign a contract that obligates the latter to display products and the former to provide incentives for sales promotion^14,20^. Whether the four major tobacco companies in South Korea―KT&G (Korea Tomorrow and Global), PMI (Philip Morris International), BAT (British American Tobacco), and JTI (Japan Tobacco International)―pay franchisors tobacco advertising allowances is unclear, as this is considered confidential business information, but these allowances are estimated to exceed 150 billion won (about 150 million U.S. dollars)^21^. By contrast, the per-store advertising allowance is 0.3–0.5 million won (about 300–500 U.S. dollars) per month^21^.

To date, the specific relationship between tobacco companies and convenience stores, as the retail POS for tobacco, has yet to be investigated. Convenience stores now play an important role in the South Korean retail landscape, especially as the consumption pattern of one-person households has changed to buying smaller quantities, and thus to shop more frequently^22^. The top two convenience store franchisors in South Korea account for more than 50% of the total market (based on the number of convenience stores)^23^, making it the third largest business in the off-line retail market, behind only hypermarkets and department stores^24^.

Therefore, the objectives of this study were to gather data on how contracts for tobacco advertisements and displays in convenience stores are made, and to investigate the relationship between tobacco companies and large retail chains. According to these results, we propose regulatory measures to prevent the further spread of tobacco products via a deepening of this relationship.

## METHODS

### Participants

In-depth interviews were conducted with three male owners (A, B, and C) of convenience stores. A list of convenience stores affiliated with these two companies was obtained for two areas, Seoul City and Gyeonggi-do (Province) in South Korea. A and C were owners of franchises of South Korea’s most famous convenience stores, and B owned a franchise belonging to the country’s second most famous convenience stores. The stores of A and B were in Seoul city, and the store of owner C store was in Gyeonggi province. Stores were randomly selected from this list, and invitations were extended to owners to participate in this study until three agreed.

### Procedures

In-depth interviews followed the process suggested by Boyce and Neale^25^. The in-depth interviews were conducted by a team of two trained male interviewers, a moderator and an assistant, who met each participant at a specified time and place. The interviews lasted about 2 hours and were conducted by the moderator according to a semi-structured framework.

### Measures and analysis

We inquired about the general status of tobacco advertising and displays at each convenience store through the questions: *‘What is the product that most affects sales?; What is the impact of selling cigarettes at convenience stores ?; What contracts exist between franchisors and tobacco companies regarding the advertising and display of tobacco products?; Do you know the total amount of the allowances that the tobacco companies paid to headquarters?; How are allowances allocated between headquarters and each franchise?; How much of the cigarette advertising allowance is allocated by the franchise headquarters?; How often is it paid: daily, monthly or annually?; How were the cigarette advertisements and displays installed in the convenience store?’.* We also asked about any additional promotional activities that were undertaken by tobacco companies after direct negotiations with individual store owners: *‘What promotional activities were conducted by tobacco companies at convenience stores?’.*

In this study, in-depth interviews were recorded on a portable voice recorder, and the data were then transcribed into Korean. All interview data were analyzed according to standard methods^25,26^. Authors iteratively read the transcribed texts, and key themes were identified and coded based on the franchisee’s responses to the questions. As data review progressed and the results were discussed by the study authors, codes were added and changed to identify key themes based on similarities. Finally, quotes representative of the key themes were selected.

## RESULTS

### Cigarettes as important merchandise

Convenience store owners reported that cigarettes are considered the most important products in these stores. Indeed, before opening a convenience store, owners seek permission to sell cigarettes because of this product’s substantial contribution to total sales.

- ‘The powerful cash cows are mainly drinks, liquor, and cigarettes. Drinks have a large margin, and liquor and cigarettes generate a lot of sales despite their small margins.’
- ‘Currently, cigarettes are the “king of sales” in convenience stores.’
- ‘The ability to sell cigarettes is very critical to the decision to operate a convenience store.’

### Contracts between franchisors and tobacco companies regarding advertising and displays

The participants reported that all of the advertisements and displays in the convenience store are dictated by a contract with the tobacco company. Such contracts specify details, such as the number of ads and the number of shelves, and are related to advertising allowances. Thus, the advertising allowance paid to the franchise is set by the tobacco company.

A. *‘What is displayed and the number of displays depend on the signed contract between franchise headquarters and the tobacco company.’*
B. *‘Cigarette advertising contracts are signed by companies. This is because space is limited, and the contract between the headquarters and each tobacco company determines the numbers of ads and displays. Each year, contracts are made based on total area; for example, Philip Morris has four rows and four columns for a total of eight units, and KT&G has two rows and seven columns for a total of 14 units.’*
B. *‘Headquarters agrees to sign a contract with the tobacco company to place the display there. The contract stipulates the following: “Two in front, one big one on top, and a cigarette showcase from here to here”. The allowance is the advertising cost, but the item is about the maintain cost of cigarette display. When I tore it off, they said, “If you do not follow the contract, we cannot pay the allowance”. If I make a contract, I have to keep my promise.’*

### Allocation of cigarette advertising allowances

As a result, when the tobacco company enters into a contract with a convenience store franchisor and pays a certain amount of money for the maintenance and display of the cigarette advertisements and showcases placed inside the convenience store, the franchise headquarters allocates a cigarette advertising allowance to the franchisee.

C. *‘I receive a monthly the advertising allowance.’*
  Franchisees receive a monthly tobacco advertising allowance from the franchisor. Currently, franchisees receive less than 1 million won (about 1000 U.S. dollars) per month, but, because this allocation is based on cigarette sales, each franchisee receives a different amount.
C. *‘I used to get paid a lot for advertising in the past. More than 1 million won a month….It has been a few years now….So now the advertising allowance does not exceed 1 million won.’*
B. *‘Advertising costs depend on the volume of tobacco sales. This is because there are shops selling lots of cigarettes, stores selling a few, and stores selling none at all.’*
C. *‘I know that it is fairly distributed.’*
  Although the sale of other products available at convenience stores, such as drinks and candy, are linked to an advertisement allowance from their headquarters, these products come in fewer varieties and are sold in lower quantities. Moreover, only cigarettes provide a consistent advertising allowance.
B. *‘There are not as many other products that steadily advertise at convenience stores. When a beverage or a candy company introduces a new product, they say, “If you post the 2+1 flyer, we will give you 5000 won”, but this lasts for only a month.’*

### Installation of cigarette advertisements and displays in convenience stores

The store owners also noted that the tobacco showcases in the convenience store were provided by the tobacco company, employees of which regularly visited and changed the displays.

A. *‘As soon as we sign a contract with headquarters, the tobacco company employees come in and place the advertising and check its placement or change their product displays.’*
B. *‘Tobacco company employees come to replace cigarettes and install new advertisements directly….I do not care, as the tobacco companies bring the cigarette showcase at their own expense. So, there’s nothing to do in the store.’*
C. *‘(A salesman) comes here once about a month…. They always carry lots of advertisements in their car.’*
  According to the franchisees, the cigarette advertisements and displays in the convenience store are not uniform but are created based on the target customer, the retail trading area, and the need to maintain consistency with their overall branding strategy.
B. *‘Ads are also placed in a unique way; small ads are placed in front of the counter… They are very concerned about this part. And they create a unified theme for each area. The Gangnam-gu area is characterized by a lot of competition with foreign tobacco companies for consumers in their 20s, so they place appropriate cigarette ads in the region. So, placement is not the same across the nation, it depends on the characteristics of the district.’*

### Convenience stores as conduits for the promotional activities of tobacco companies

The promotional activities of each convenience store vary depending on the salesperson sent by the tobacco company, and some convenience store owners reported receiving an additional incentive in the form of payments for sales promotions to increase product sales. Such incentives can be based on the volume of tobacco sales or the failure to sell competitors’ products.

C. *‘That’s the difference between salespeople. There are salesmen who do not care.’*
C. *‘This was also the case. The salesperson said, “I will multiply the number of sales between Sept. 1 and Sept. 30 by 100 won and give it to you in cash”.’*
B. *‘They promote illegal sales….They said, “we will give you a subsidy instead….Every time you do not sell this product, we’ll give you some money”. If I do not sell a cigarette, I lose income. So, they give me enough to make up for it.’*
  Additionally, when a new product is launched, free samples are offered for promotional purposes, and the tobacco company sends a part-time worker directly to the convenience store to promote the product.
A. *‘When the new cigarette was released, the salesperson gave me a pack to test the taste.’*
C. *‘When I place this cigarette pack here, customers know that “It’s free or It’s a demonstration”. When they ask, “What is this?” we say, “You can try it”.’*
C. *‘In the past, they used to hire part-time workers….The tobacco company hired a part-time worker and sent him directly to a convenience store. The part-time worker recommended a product at a convenience store, saying, “Would you like to try this new product?”.’*

## DISCUSSION

**Figure 1:**
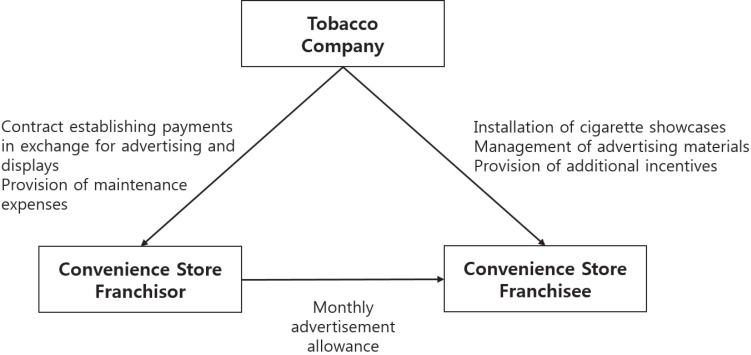
Advertising-related relationships among tobacco companies, franchisors, and franchisees

The purpose of this study was to gather data on the advertising and display status of tobacco products at convenience stores, the major sites of retail tobacco sales, by interviewing convenience store owners. We found that the characteristics of cigarette advertising and displays, such as quantity, location, display method, type of advertisement, and so on, are determined by a contract between the tobacco companies and the convenience store headquarters. Additionally, the franchise headquarters receives an advertising allowance from the tobacco company, and the franchisee is paid a monthly allowance by the franchisor to maintain the advertisement and display of tobacco products. As specified in contractual agreements with retail chains, tobacco companies install tobacco advertisements and displays in stores; however, they also engage in illegal promotional activities (Figure 1).

Selling tobacco products is one of the most financially beneficial activities in the retail sector^27,28^. South Korean owners recognize the importance of tobacco products, as cigarette sales account for 30–40% of the total gross sales of convenience stores, and thus directly affect total profits^29^. Additionally, the cigarette advertising allowance is tied to the sale of tobacco products. According to the contract between the convenience store franchise and the franchisee^29-31^, the franchisor provides support and education regarding management and sales activities as well as initial stabilization grants, long-term operating grants, and sales incentives to ensure that the economic interests of the franchise are maintained by the franchisee. The cigarette advertising allowance is a fixed supplementary income provided to each convenience store franchisee from the monthly subsidy received by headquarters.

Globally, tobacco companies spend tens of billions of dollars on TAPS each year^32^. In-store advertising expenditures have been increasing as other tobacco control regulations have tightened^33^. In 2016, a total of $9.5 billion were spent on adverting and promotion by the cigarette companies ($8.7 billion) and smokeless tobacco companies ($0.8 billion) in the United States^34,35^. In particular, a substantial amount of expenditure was paid to cigarette retailers for price discounts ($5.8 billion) or promotional allowances ($0.2 billion)^34^.

Clearly, if this subsidy was eliminated due to the prohibition of advertisements and displays, franchisors and franchisees would undoubtedly express strong opposition. It may be difficult to enforce regulations without support from the headquarters of convenience stores. In fact, in 2014, the South Korean government announced a comprehensive tobacco control plan to reduce the demand for tobacco^36^; this plan included increasing the price of tobacco products, introducing graphic health warning labels, and banning tobacco ads in POS. The stores expressed their concerns about a potential decrease in their allowances^37^. In 2013, the operators of the 7–Eleven convenience stores even filed a lawsuit against their headquarters, involving a demand by franchisees for a greater advertising allowance^29,38^. Although the court ruled against the plaintiffs because they had, in fact, received an advertising allowance, the franchisees continue to feel entitled to an increase in this allowance. Therefore, financial support may be considered when banning tobacco advertising and displays at POS^20,39^. Ultimately, it is necessary to build a retail market that can operate without relying on tobacco advertising allowances.

As in other countries^40-42^, the Korean government’s plan to introduce graphic health warnings on tobacco packaging was strongly opposed by the Korean Association of Convenience Stores, which claimed that it would cause a loss of profits and thus adversely affect the mental health of convenience store customers and workers^43^. However, retailers and tobacco companies have long had a close relationship, the existence of which is widely recognized^14,20^. The contracts behind these relationships do not involve merely setting up and paying for the installation of cigarette advertising in convenience stores. The tobacco lobby, through promotional allowances^44^, has contributed funds to organizations not directly related to tobacco, including retailer groups, restaurant associations, and the hospitality industry, with the aim of weakening tobacco control regulations. Moreover, the list of organizations receiving allowances is expanding to other industrial and commercial sectors^45-47^. Indeed, the tobacco industry has attempted to maintain wider relationships with the distribution industry, especially by expanding its retail chain market, which makes the distribution industry financially dependent on the tobacco industry^45-47^. This can be seen as a strategy by tobacco companies to secure a front group that can influence the tobacco control legislative process^48^ by financially supporting it. Thus, it may be difficult to enforce tobacco control regulations without initially obtaining the cooperation of the front groups established by the tobacco industry, such as convenience stores. Therefore, to prepare for class actions and other forms of opposition that delay implementation of such regulations, it is necessary to collect and analyze data from all stakeholders before introducing tobacco-related regulations.

Furthermore, by implementing a ban on tobacco advertising and displays at POS, the government should also prepare for litigation, because the multinational tobacco industry has consistently used legal challenges to prevent the enactment of tobacco control regulations^49^, arguing that there is no scientific evidence to prove that smokers continue to smoke and that non-smokers start smoking as a result of the advertising and displays at POS^50^. Rather, as these are intended as product information for smokers, banning these materials violates rights of freedom of expression and free enterprise^50^. However, as the FCTC stipulates, a ban on tobacco advertising at POS is a non-price policy that has been credibly proven to be effective^8^.

Moreover, in Norway, Panama, Columbia, and Costa Rica, restricting the interests of tobacco companies through the ban of tobacco advertising was deemed constitutional because it is a necessary measure to protect public health and the ban does not interfere with free trade^49^. These cases have made it more difficult for the tobacco industry to succeed in overturning bans on cigarette advertising and displays at POS in other countries. In fact, Iceland, Canada, Thailand, Australia, Ireland, UK, New Zealand and Norway are among the countries that have banned cigarette advertising at POS^51^. The introduction of such measures elsewhere around the world, therefore, seems likely^52^.

Following the enactment of the National Health Promotion Act in 1995, South Korea has implemented a variety of tobacco control policies, including extending smoking ban areas, providing and promoting prevention education programs directed at the public, and supporting smoking cessation^53^. As a result, there has been a dramatic short-term decrease in the prevalence of smoking among adult males aged 15 years and older, from 64.1% in 2000^54^ to 48.5% in 2013^55^. However, the decreased tobacco sales due to the decline in smoking prevalence has encouraged competition among the tobacco companies. Specifically, in 1989, a global tobacco company was allowed to enter the domestic market and, in 2001, the privatization of KT&G led to competition between the domestic tobacco company and a global tobacco company for the domestic tobacco market^56^.

In addition, because tobacco advertising through mass media is prohibited, the legal and consistently available advertising venues for tobacco companies are convenience stores, in which they can place advertisements and displays. In this context, we expected to find that tobacco companies engaged in promotional activities other than placing advertisements and displays at convenience stores^57^. Despite its focus on only a few cases, this research determined that tobacco companies do, in fact, engage in complex illegal promotional activities. Although the placement of advertisements and displays at convenience stores follows strict contractual agreements, the sales representatives of tobacco companies engage in illegal promotional activities in stores with which their companies have individual contacts. At this time, tobacco companies not only provide incentives to franchisees^14,20^ but also verbally communicate with them directly to promote their products. Although retailers are aware that certain promotional activities are illegal, they nonetheless accept the financial rewards offered by the tobacco companies for engaging in these activities.

Based on our findings, we recommend that vendors be asked to voluntarily refrain from participating in illegal promotional activities^39,58^ and that appropriate education be provided to tobacco sellers^59^. Although the Ministry of Strategy and Finance has the authority to prohibit the offering of money, goods, etc., for the promotion of tobacco sales, there is no action or measure allowing for the monitoring of these illegal activities. However, they could be identified and monitored through various types of research and activities aimed at continuously disclosing such practices. Tobacco sellers could be made aware that they are being monitored and that public attention is being drawn to this issue^60,61^.

This study has several limitations. First, the number of participating franchise owners was very small (n=3); indeed, because franchisees have an obligation not to disclose management information, it was difficult to recruit representative participants. Despite our contacting many owners, none from the third largest franchise chain agreed to participate in our study. However, as the operations of convenience stores are standardized by the individual franchise brands^62^, we believe that the three participants were able to provide representative information, as the contracts between tobacco companies and convenience store franchises would have been the same even if more owners had been interviewed. Second, it was difficult to generalize the results on the promotional activities of tobacco companies. Although we found that illegal promotional activities differed according to the target customers and retail trade areas, we had few examples, and differences between regions and chain stores could not be determined. Further interviews and surveys should be conducted to identify potential differences among locations, regions, and franchise chains.

## CONCLUSIONS

The sale and display of tobacco products at convenience stores ‘normalizes’ tobacco products by treating them as common and essential, similar to other products^63,64^; this promotes continued smoking and creates new smokers. Therefore, to ‘denormalize’ smoking and phase out tobacco products throughout society, we suggest that tobacco advertisements and displays in convenience stores be banned as soon as possible. The expansion of convenience stores is related to the increase in tobacco advertisements and displays at POS. Tobacco products not only directly affect the total profits of convenience stores, they also provide additional income in the form of monthly payments in return for advertising. For these reasons, convenience stores continue to feature cigarette advertising. Tobacco companies also provide financial incentives to franchisees for participating in illegal promotional activities. Promotional activities should be periodically monitored to detect those that are illegal and to limit tobacco sales and advertising. Comprehensive data from all stakeholders should be collected and analyzed before bans on advertising and displays at POS are introduced.
